# Supplementary material for: A Free-Standing Polymer Polypyrrole/Cellulose Composite Film via Spatial-Confined Interfacial Electrodeposition for Flexible Supercapacitors
Source: Materials (Basel). 2023 Sep 29;16(19):6476. doi: 10.3390/ma16196476 (PMC10573258; doi:10.3390/ma16196476)
Supplement: Supplementary file 1 [file materials-16-06476-s001.zip › materials-2602092-supplementary.pdf]

Supporting information

A Free-Standing Polymer Polypyrrole/Cellulose Composite Film via Spatial-Confined Interfacial Electrodeposition for Flexible Supercapacitors

Sijie Wang [1], Wen Chen [1], Xinyue Huang [1], Xuezheng Chen [1], De Li [1], Feng Yu [1,*] and Yong Chen [2,*]

[1] State Key Laboratory of Marine Resource Utilization in South China Sea, Hainan Provincial Key Laboratory of Research on Utilization of Si-Zr-Ti Resources, Hainan University, Haikou 570228, China; wwsj1207@163.com (S.W.); chenwen996@126.com (W.C.); huangxinyue122@163.com (X.H.); c18760162082@163.com (X.C.); lidenju@sina.com (D.L.)

[2] Guangdong Key Laboratory for Hydrogen Energy Technologies, School of Materials Science and Hydrogen Energy, Foshan University, Foshan 528000, China

* Correspondence: yuf@hainanu.edu.cn (F.Y.); ychen2002@163.com (Y.C.)

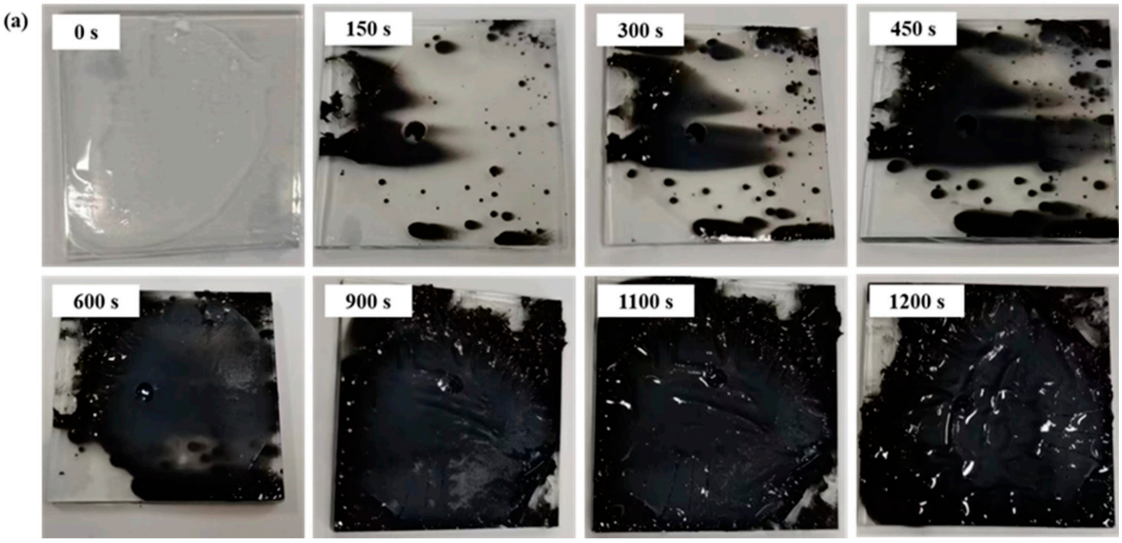

Figure S1. Process diagram of PPy/cellulose film

(a) (b)

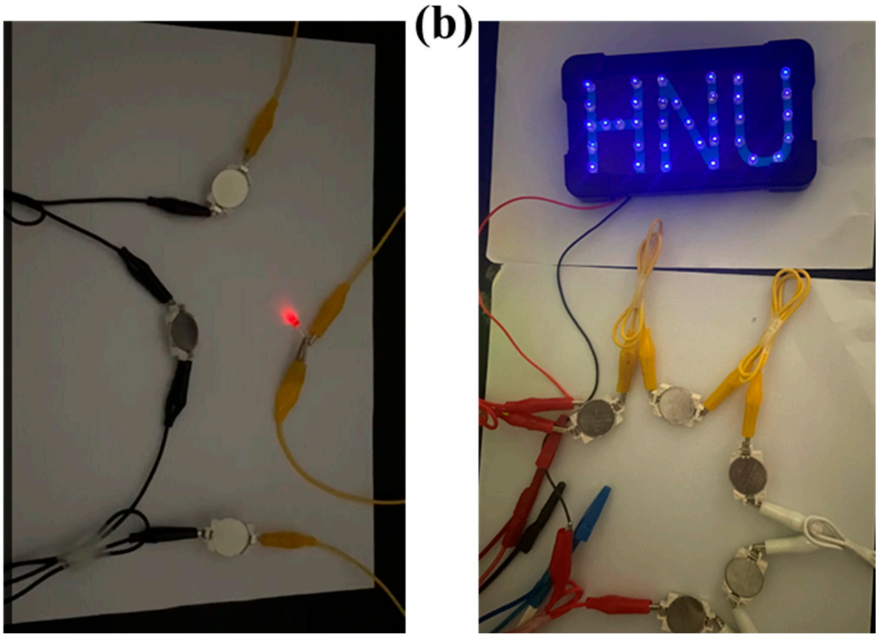

Figure S2. The prepare device lights a small bulb (a) and an electronic lamp board (b)
